# Mutation and Selection Cause Codon Usage and Bias in Mitochondrial Genomes of Ribbon Worms (Nemertea)

**DOI:** 10.1371/journal.pone.0085631

**Published:** 2014-01-15

**Authors:** Haixia Chen, Shichun Sun, Jon L. Norenburg, Per Sundberg

**Affiliations:** 1 Department of Biological and Environmental Sciences, University of Gothenburg, Sweden; 2 Institute of Evolution & Marine Biodiversity, Ocean University of China, Qingdao, China; 3 Department of Invertebrate Zoology, Smithsonian's National Museum of Natural History, Washington D.C., United States of America; Tel Aviv University, Israel

## Abstract

The phenomenon of codon usage bias is known to exist in many genomes and it is mainly determined by mutation and selection. To understand the patterns of codon usage in nemertean mitochondrial genomes, we use bioinformatic approaches to analyze the protein-coding sequences of eight nemertean species. Neutrality analysis did not find a significant correlation between GC12 and GC3. ENc-plot showed a few genes on or close to the expected curve, but the majority of points with low-ENc values are below it. ENc-plot suggested that mutational bias plays a major role in shaping codon usage. The Parity Rule 2 plot (PR2) analysis showed that GC and AT were not used proportionally and we propose that codons containing A or U at third position are used preferentially in nemertean species, regardless of whether corresponding tRNAs are encoded in the mitochondrial DNA. Context-dependent analysis indicated that the nucleotide at the second codon position slightly affects synonymous codon choices. These results suggested that mutational and selection forces are probably acting to codon usage bias in nemertean mitochondrial genomes.

## Introduction

The genetic code is degenerate (64 codons for 20 amino acids and the termination signal), with most amino acids encoded by two to six synonymous codons. In protein-coding genes, synonymous codons that code for the same amino acid do not appear at the same frequency [1], [2]. Bias in synonymous codon usage exists in a wide variety of organisms, from prokaryotes, to unicellular and multicellular eukaryotes [3], [4], [5]. Since different genomes have their own characteristic patterns of synonymous codon usage [6], it has not been easy to provide a satisfactory explanation for the particular pattern that is found in a given genome. In some prokaryotes, such as *E. coli*, and some unicellular eukaryotes, e.g., yeast, codon usage is thought to be determined by the equilibrium between natural selection and mutation bias [7], [8], [9], [10]. Mutation bias is the major factor accounting for the variation in codon usage in some prokaryotes with extremely high A + T or G + C contents [11], and in many mammals [12]. However, translational selection plays the most important role in shaping codon usage in *Drosophila* [13], and in some plants [14], [15], [16].

Analysis of codon usage facilitates the understanding of evolution and environmental adaptation of living organisms [17]. While numerous reports on synonymous codon usage bias have focused on nuclear genomes, only few mitochondrions have been analyzed. Mitochondrial genomes are an evolutionary paradox with a relatively conserved gene content and small size. The genetic code of mitochondria furthermore often differs from the standard genetic code [18]. We know that the codon usage pattern changes during evolution, but how is still not completely known. Mitochondrial codon usage has been studied mainly in vertebrates, whereas among invertebrates so far only some parasitic platyhelminthes had been surveyed for mitochondrial codon usage and bias [19]. Currently, the complete or nearly complete mitochondrial genomes of nine nemerteans have been sequenced. These species include two palaeonemertans (*Cephalothrix hongkongiensis*, *C. rufifrons*, *C*. sp.), three heteronemerteans (*Lineus alborostratus*, *L. viridis*, *Zygeupolia rubens*), and three hoplonemerteans (*Emplectonema gracile*, *Nectonemertes* cf. *mirabilis*, *Paranemertes* cf. *peregrina*) (e.g., [20], [21], [22], [23], [24]). Thus all three major lineages of nemerteans are represented and it is now possible to investigate codon usage bias and associated forces in phylum Nemertea. Knowledge of codon patterns in the different nemerteans should improve the understanding of the mechanism of codon distribution and variation in nemertean mitochondrial genomes, and of factors shaping codon usage patterns. In this paper, we report the analysis of codon usage bias in eight nemertean mitochondrial genomes by using methods of multivariate statistical analysis and correlation analysis, and we also compare codon usage and tRNAs.

## Materials and Methods

### Data retrieval

Except for *Cephalothrix rufifrons*, for which we lack complete data for protein-coding genes, the complete or nearly complete mitochondrial genome sequences from *Cephalothrix hongkongiensis* (NC_012821), *Cephalothrix* sp. (NC_014869), *Lineus alborostratus* (NC_018356), *L. viridis* (NC_012889), *Zygeupolia rubens* (NC_017877), *Emplectonema gracile* (NC_016952), *Nectonemertes* cf. *mirabilis* (NC_017874), and *Paranemertes* cf. *peregrina* (NC_014865) were obtained from GenBank and all protein-coding sequences from each genome were retrieved.

### General aspects of nemertean mitogenomes

GC content of the entire gene, first, second, and third codon positions (GCall, GC1, GC2, and GC3 respectively) were calculated after excluding the stop codons. GC12 is the average of GC1 and GC2, and was used for analysis of neutrality plots (GC12 *vs* GC3 [25]). To investigate the nucleotide bias, skew was calculated as (A−T)/(A+T) or (G−C)/(G+C) [26]. Relative synonymous codon usage (RSCU) values were calculated with MEGA 5 [27]. RSCU values greater than 1.0 indicate that the corresponding codons are used more frequently than the expected frequency whereas the reverse is true for RSCU values less than 1.0.

### Codon usage bias: measured by ENc

Effective number of codons (ENc) [28] is a widely used measure of codon bias. It ranges from 20 (in an extreme case, only one codon for each codon family was used) to 61 (all synonymous codons were equally used). ENc value was calculated with DAMBE 5 [29] for both combined coding sequences and individual genes. The ENc-GC3s plot has been widely used to determine whether codon usages of given genes are affected by mutation only (corresponding points would lie around the expected curve) or also by other factors such as selection (corresponding points would depart away from, considerably below the expected curve).

At individual gene level, ENc calculation is sometimes inaccurate due to statistical fluctuation and internal mathematical flaws, as pointed out by Fuglsang [30]. For this reason, only those protein-coding gene sequences that were 200 codons or more in length were used to draw ENc-GC3s plots.

### PR2-bias plot analysis

The Parity Rule 2 (PR2) bias is detected by the value of AT-bias [A/(A+ T)] as the ordinate and GC-bias [G/(G + C)] as the abscissa[31]. In this plot, the center of the plot, where both coordinates are 0.5, is the place where A =  T and G  = C (PR2), with no biases between the two complementary strands of DNA in mutation and selection rates (substitution rates). A vector from the center represents the extent and direction of biases from PR2. PR2 bias plots are particularly informative when PR2 biases at the third codon position in the four-codon amino acids of individual genes are plotted [32]. In this case, ‘A3/(A3 + T3)’ and ‘G3/(G3+C3)’ are plotted as the ordinate and abscissa, respectively.

### Statistical tests for context-dependent codon usage bias

Methods previously applied to animal mitogenomes [33] and plant mitogenomes [34] were adopted to detect the influences on codon usage by context-dependent mutation in each nemertean mitochondrion. To avoid the constraint from amino acid usage, only data of the four-fold degenerate (FFD) families (Ala, Arg, Gly, Leu, Pro, Ser1, Ser2, Thr, Val) were used. The null hypothesis is that mutations are independent single-site events; therefore, the base frequencies of the third codon position in FFD families are not influenced by bases at the second codon positions. To test the independence between the second and third codon positions, the following three datasets were used: (i) Leu(CUN)/Pro(CCN)/Arg(CGN); (ii) Val(GUN)/Ala(GCN)/Gly(GGN); and (iii) (Leu+Val, CUN+GUN)/(Ser1+Pro+Thr+Ala, NCN)/(Arg+Gly+ Ser2, CGN+GGN+AGN). For each dataset and species, *x* ^2^ independence test with 6 degrees of freedom was conducted. The final results were then organized and compared across datasets and species.

### Statistical analysis

Statistically significant (*P*<0.05) associations were tested using χ^2^ test and correlations were analyzed using the Spearman's rank correlation test. GraphPad Prism 5 software (GraphPad Software, San Diego, CA, USA) was used for statistical evaluation.

## Results and Discussion

### GC content variation in nemertean mitochondrial genomes

**Figure 1:**
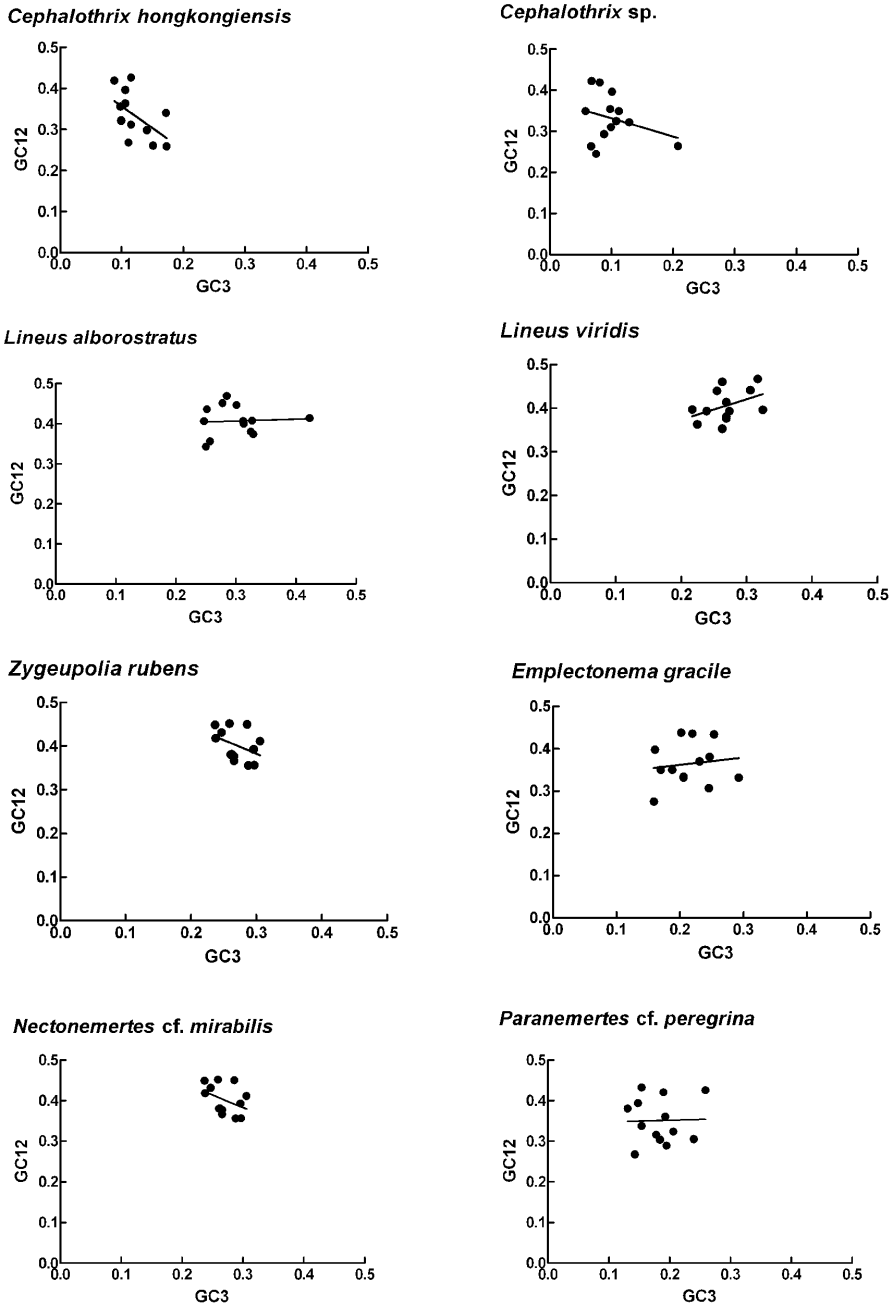
Neutrality plot (GC12 against GC3). *Cephalothrix hongkongiensis*; the regression line is y = −1.0593x+0.4625, R^2^ = 0.2862. *Cephalothrix* sp.; the regression line is y = −0.4401x+0.3755, R^2^ = 0.0857. *Lineus alborostratus*; the regression line is y = 0.4684x+0.2799, R^2^ = 0.1746. *Lineus virids*; the regression line is y = 0.4684x+0.2799, R^2^ = 0.1746. *Zygeupolia rubens*; the regression line is y = −0.6239x+0.5701, R^2^ = 0.1583. *Emplectonema gracile*, the regression line is y = 0.3281x+0.1697, R^2^ = 0.0173. *Nectonemertes* cf. *mirabilis*; the regression line is y = 0.4506x+0.2396, R^2^ = 0.0968. *Paranemertes* cf. *peregrina*; the regression line is y = 0.0422x+0.3429, R^2^ = 0.0008.

Table 1 summarizes nucleotide composition of the analysed mitochondrial genomes. All the protein-coding genes of the eight species are transcribed in the same direction (L strand). Among the eight species, the genomic GC contents (GCall) varied from 26.6% in *Cephalothrix* sp. to 37.2% in *Lineus alborostratus*. The GC1, GC2 and GC3 are low in the eight nemertean mitochondrial genomes, which is consistent with the generally lower genomic GC contents in invertebrate mitochondrial genomes. The GC3s are significantly higher in heteronemerteans than those in hoplonemerteans and palaeonemerteans, as well as GC1 and GC2 values. Furthermore, the GC3s are significantly lower than GC1 and GC2 (*P*<0.05). Differences in GC content are greatest at the third codon position followed by the first and second positions. The neutrality plots (GC12 *vs* GC3) (Figure 1) show that all eight nemertean mitochondrial genomes had narrow GC3 distributions. Except for *C*. *hongkongiensis* (*r* = −0.557, *P*<0.05), there were no significant correlations (*r* = −0.535, −0.049, 0.032, 0.318, −0.492, 0.131, 0.318, −0.028, *P* > 0.05 in all cases) in the other seven nemerteans, suggesting low mutation bias or high conservation of GC content level throughout the whole genome.

**Table 1:** Summary of codon usage in the seven nemertean mitochondrial genomes.

Species	Classification	Genome size (bp)	Protein coding gene	GC all (%)	GC1(%)	GC2(%)	GC3(%)	AT skew	GC skew	ENc
*Cephalothrix hongkongiensis*	Paleonemertea	16,296	13	26.6	34.6	33.8	11.4	−0.27	0.19	33.7
*Cephalothrix* sp.	Paleonemertea	15,800	13	25.5	34.2	33.5	8.7	−0.27	0.18	32.5
*Lineus alborostratus*	Heteronemertea	15,476	13	37.2	43.6	37.7	30.1	−0.35	0.31	51.0
*Lineus viridis*	Heteronemertea	15,388	13	35.9	43.1	37.8	26.8	−0.27	0.29	39.5
*Zygeupolia rubens*	Heteronemertea	15,513	13	35.8	44.0	36.8	26.6	−0.36	0.42	35.9
*Emplectonema gracile*	Hoplonemertea	14,666	13	32.0	39.5	34.9	21.7	−0.44	0.22	39.6
*Nectonemertes* cf. *mirabilis*	Hoplonemertea	15,365	13	31.3	36.7	33.6	23.6	−0.38	0.29	36.8
*Paranemertes* cf. *peregrina*	Hoplonemertea	14,558	13	30.1	37.4	34.4	18.4	−0.35	0.32	35.6

### Codon bias level of nemertean mitochondrial genes: measured by ENc

**Figure 2:**
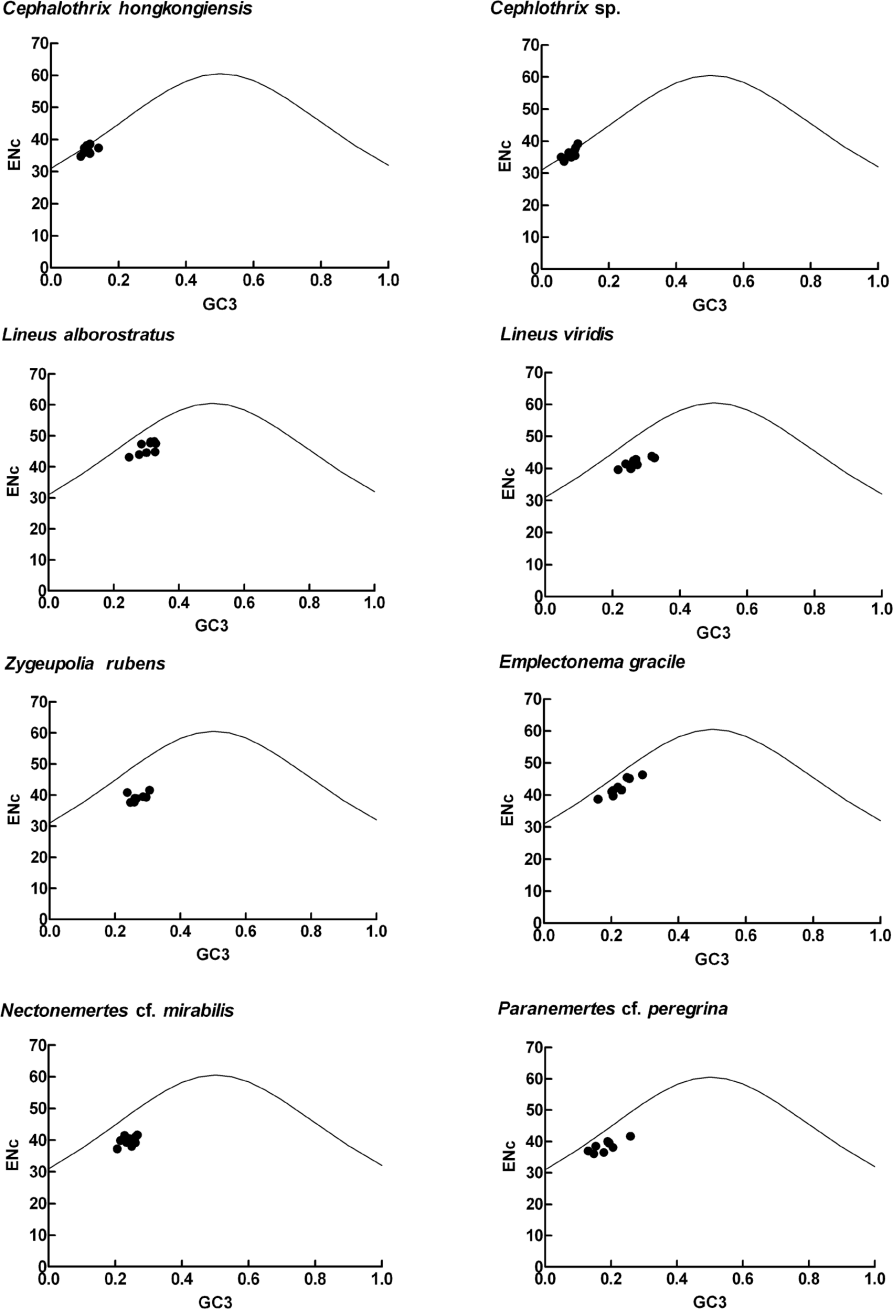
Relation between GC3 and ENc (ENc-plot). ENc were plotted against GC content at the third codon position. The expected ENc from GC3 are shown as a standard curve.

The effective number of codons (ENc) has been widely used to measure the codon bias level of individual genes [28]. The more biased a gene, the smaller the ENc value. In all eight mitochondrial genomes, ENc values of all individual genes (>600 bp) were calculated. Table 2 lists ENc values of 9 mitochondrial genes (except *atp8*, *nad4L*, *nad3*, *nad6*) from all eight genomes. For each gene, the ENc value is the highest in *L*. *alborostratus*; in contrast, the values are lowest in two *Cephalothrix* species. ENc-plot (Figure 2) of eight nemerteans showed that although there were a small number of genes close to the expected ENc-plot curve, which indicates that mutation plays a role in defining the codon usage variation among those genes, a majority of the points are below the expected curve. This suggests that not only mutation but also other factors, such as translational selection, are likely to be involved in determining the selective constraints on codon bias in nemertean mitochondrial genomes.

**Table 2:** ENc values of 9 mitochondrial genes.

Species	*atp6*	*cob*	*cox1*	*cox2*	*cox3*	*nad1*	*nad2*	*nad4*	*nad5*
*Cephalothrix hongkongiensis*	30.86	32.61	28.89	29.95	30.15	32.95	28.98	31.74	31.12
*Cephalothrix* sp.	30.92	34.45	28.9	30.62	29.61	30.81	28.74	29.95	30.03
*Lineus alborostratus*	42.66	41.65	44.57	47.52	41.49	49.85	44.77	46.35	47.0
*Lineus viridis*	39.64	35.56	37.35	34.92	42.08	38.7	41.31	42.24	40.55
*Zygeupolia rubens*	33.46	39.73	32.86	31.12	32.96	36.22	34.46	32.12	36.89
*Emplectonema gracile*	46.02	36.54	37.84	38.76	40.98	39.04	35.34	48.24	35.69
*Nectonemertes* cf. *mirabilis*	35.74	35.37	36.73	35.58	34.66	38.2	39.74	37.1	35.26
*Paranemertes* cf. *peregrina*	29.79	33.3	37.16	37.86	33.75	35.92	33.51	36.27	36.29

### Base composition bias and codon usage bias

The AT skews for the eight nemertean mitochondrial genomes are all negative, while GC skews are all positive. This indicates asymmetry in nucleotide composition between the two strands, with one being rich in A and C, and the other being rich in T and G (Table 1). This phenomenon is common in mitochondrial genomes [26], and suggests the presence of asymmetric patterns of mutational changes between strands [31], [35].

RSCU analysis unraveled a general bias toward codons having nucleotides A or U at the third position and U was detected more frequently (Table 3), similar to what has been found in some other invertebrate mitochondrial genomes [19]. These findings may indicate directional mutation in the codon usage patterns of nemertean mitochondrial genomes, and suggest the correlation between genome A+T content and overall codon bias, which is consistent with vertebrate mitogenomes [36].

**Table 3:** Comparison of RSCU and synonymous codon families (SCF) of eight nemerteans.

tRNA	amino acid	Anticodon	SCF	Codon	Ch§	C.sp	La	Lv	Zr	Eg	Nm	Pp	average
Glu	**E**	**UUC**	**GAR**	**GAA**	1.44	1.54	1.35	0.91	0.83	1.06	0.82	0.9	1.11
	**E**			GAG	0.56	0.46	1.19	1.09	1.17	0.94	1.18	1.1	0.96
													
Lys	**K**	**UUU**	**AAR**	**AAA**	1.59	1.74	1.91	1.17	0.91	1.59	1.21	1.24	1.42
	**K**			AAG	0.41	0.26	0.81	0.83	1.09	0.41	0.79	0.76	0.67
													
Leu	**L**	**UAA**	**UUR**	**UUA**	3.82	4.34	4.83	2.12	2	2.56	2.38	3.24	3.16
	**L**			UUG	1.17	0.79	4.56	1.97	2.8	1.4	1.76	1.44	1.99
													
Trp	**W**	**UCA**	**UGR**	**UGA**	1.69	1.68	1.65	1.03	0.62	1.3	0.87	0.97	1.23
	**W**			UGG	0.31	0.32	1.46	0.97	1.38	0.7	1.13	1.03	0.91
													
Gln	**Q**	**UUG**	**CAR**	**CAA**	1.56	1.67	0.78	0.78	0.44	1.16	0.54	1.04	1.00
	**Q**			CAG	0.44	0.33	0.86	1.22	1.56	0.84	1.46	0.96	0.96
													
Met	**M**	**CAU**	**AUR**	AUA	1.36	1.49	0.95	0.94	0.68	1.01	0.94	1.31	1.09
	**M**			**AUG**	0.64	0.51	1.05	1.06	1.32	0.99	1.06	0.69	0.92
													
Cys	**C**	**GCA**	**UGY**	**UGC**	0.12	0.2	0.3	0.19	0.08	0.33	0.29	0.17	0.21
	**C**			UGU	1.88	1.8	1.73	1.81	1.92	1.67	1.71	1.83	1.79
													
Asp	**D**	**GUC**	**GAY**	**GAC**	0.16	0.11	0.32	0.29	0.07	0.35	0.26	0.08	0.21
	**D**			GAU	1.84	1.89	1.86	1.71	1.93	1.65	1.74	1.92	1.82
													
Phe	**F**	**GAA**	**UUY**	**UUC**	0.13	0.12	0.40	0.09	0.04	0.22	0.18	0.08	0.16
	**F**			UUU	1.87	1.88	0.94	1.91	1.96	1.78	1.82	1.92	1.76
													
His	**H**	**GUG**	**CAY**	**CAC**	0	0.08	0.40	0.3	0.18	0.31	0.2	0.19	0.21
	**H**			CAU	2	1.92	1.65	1.7	1.92	1.69	1.8	1.81	1.81
													
Ile	**I**	**GAU**	**AUY**	**AUC**	0.11	0.08	0.18	0.21	0.02	0.17	0.03	0.05	0.11
	**I**			AUU	1.89	1.92	1.82	1.79	1.98	1.83	1.97	1.95	1.89
													
Asn	**N**	**GUU**	**AAY**	**AAC**	0.18	0.17	0.78	0.35	0.09	0.33	0.11	0.14	0.20
	**N**			AAU	1.82	1.92	1.48	1.65	1.91	1.67	1.89	1.86	1.78
													
Tyr	**Y**	**GUA**	**UAY**	**UAC**	0.2	0.23	0.65	0.44	0.08	0.4	0.16	0.26	0.30
	**Y**			UAU	1.8	1.77	3.05	1.56	1.92	1.6	1.84	1.74	1.91
													
Ala	**A**	**UGC**	**GCN**	**GCA**	0.61	0.67	0.77	0.55	0.64	0.6	0.26	0.62	0.59
	**A**			GCC	0.15	0.14	0.49	0.26	0.11	0.33	0.32	0.05	0.23
	**A**			GCG	0.02	0.07	0.77	0.6	0.32	0.3	0.52	0.32	0.37
	**A**			GCU	3.22	3.11	1.97	2.58	2.93	2.77	2.91	3	2.81
													
Gly	**G**	**UCC**	**GGN**	**GGA**	1.53	1.76	0.82	0.69	0.63	1.28	0.66	1.18	1.07
	**G**			GGC	0.03	0.06	0.32	0.24	0.09	0.2	0.27	0.06	0.16
	**G**			GGG	0.36	0.14	1.48	1.48	1.52	1.02	1.48	1.22	1.09
	**G**			GGU	2.09	2.04	1.39	1.6	1.76	1.49	1.59	1.55	1.69
													
Leu	**L**	**UAG**	**CUN**	**CUA**	0.25	0.19	0.28	0.21	0.11	0.31	0.25	0.14	0.22
	**L**			CUC	0.03	0.01	0.13	0.04	0.02	0.16	0.12	0.03	0.07
	**L**			CUG	0.06	0.03	0.44	0.19	0.14	0.19	0.19	0.09	0.17
	**L**			CUU	0.66	0.63	1.36	1.47	0.93	1.38	1.30	1.05	1.10
													
Pro	**P**	**UGG**	**CCN**	**CCA**	0.43	0.6	0.49	0.31	0.54	0.56	0.48	0.47	0.49
	**P**			CCC	0.16	0.13	0.52	0.37	0.14	0.32	0.32	0.11	0.26
	**P**			CCG	0.03	0	0.7	0.54	0.37	0.39	0.53	0.15	0.34
	**P**			CCU	3.38	3.27	2.3	2.78	2.95	2.74	2.67	3.27	2.92
													
Arg	**R**	**UCG**	**CGN**	**CGA**	1.41	1.45	1.28	0.6	1.14	1.35	0.51	1.33	1.13
	**R**			CGC	0.12	0	0.28	0.22	0.1	0.25	0.11	0.12	0.15
	**R**			CGG	0.59	0.17	0.83	1.1	0.83	0.62	1.26	0.73	0.77
	**R**			CGU	1.88	2.38	1.61	2.08	1.92	1.78	2.11	1.82	1.95
													
Ser1	**S**	**UGA**	**UCN**	**UCA**	1.06	1.15	0.64	0.27	0.28	0.55	0.32	0.52	0.60
	**S**			UCC	0.22	0.21	0.25	0.22	0	0.26	0.26	0.15	0.20
	**S**			UCG	0.07	0	0.27	0.36	0.4	0.26	0.3	0.17	0.23
	**S**			UCU	4.25	4.03	2.63	3.51	3.44	4.06	4.13	3.85	3.74
													
Thr	**T**	**UGU**	**ACN**	**ACA**	0.51	0.65	0.85	0.33	0.64	0.71	0.55	0.85	0.64
	**T**			ACC	0.13	0.03	0.31	0.11	0.08	0.5	0.05	0.04	0.16
	**T**			ACG	0.03	0	0.42	0.4	0.52	0.21	0.14	0.43	0.27
	**T**			ACU	3.33	3.32	2.42	3.16	2.76	2.58	3.27	2.68	2.94
													
Val	**V**	**UAC**	**GUN**	**GUA**	1.06	1.31	0.58	0.48	0.46	0.57	0.39	0.62	0.68
	**V**			GUC	0.07	0.07	0.31	0.28	0.07	0.25	0.13	0.11	0.16
	**V**			GUG	0.06	0.09	0.96	0.72	0.68	0.64	0.67	0.63	0.56
	**V**			GUU	2.82	2.53	2.15	2.52	2.79	2.54	2.81	2.65	2.60
													
Ser2	**S**	**KCU** *	**AGN**	**AGA**	0.99	1.31	1.36	0.98	1.06	0.89	1.12	0.63	1.04
	**S**			**AGC**	0.07	0.05	0.57	0.4	0.07	0.14	0.14	0.06	0.19
	**S**			AGG	0.36	0.18	1.16	0.89	1.34	0.69	0.97	0.75	0.79
	**S**			AGU	0.99	1.06	1.65	1.38	1.41	1.15	1.46	1.16	1.28

Ch:Cephalothrix hongkongiensis; C.sp.:Cephalothrix sp.; La: Lineus alborostratus; Lv: Lineus viridis; Zr:Zygeupolia rubens; Eg:Emplectonema gracile; Nm:Nectonemertes cf. mirabilis; Pp:Paranemertes cf. peregrina.

The anticodons of tRNA-Ser are GCT in Cephalothrix hongkongiensis, C. sp., Emplectonema gracile, Nectonemertes cf. mirabilis, Paranemertes cf. peregrina, but TCT in Lineus alborostratus, L. viridis, Zygeupolia rubens.

**Figure 3:**
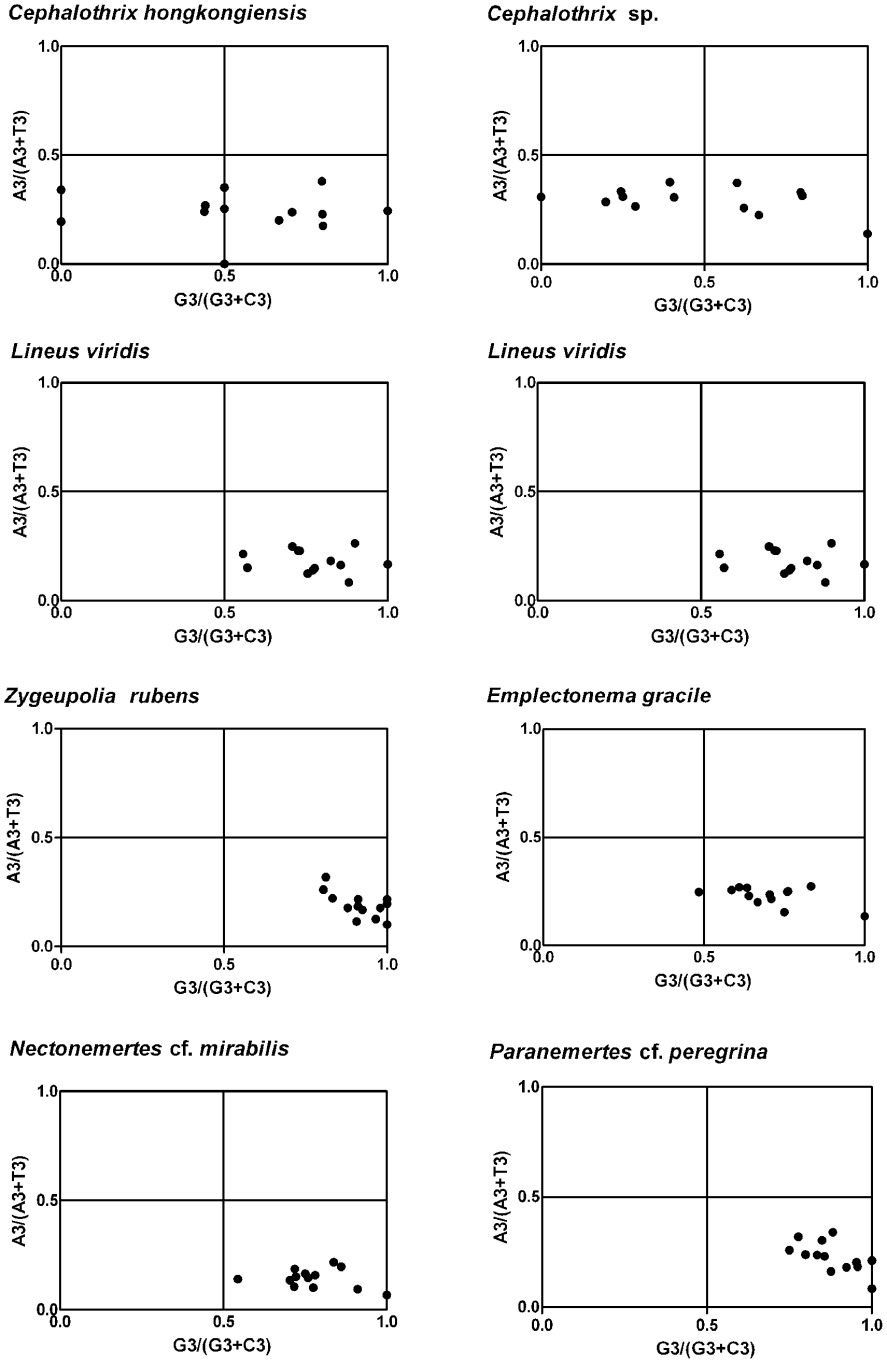
PR2-bias plot [A3/(A3 + T3) against G3/(G3+ C3)]. *Cephalothrix hongkongiensis*; average position is x = 0.2396±0.0951, y = 0.5503±0.2980. *Cephalothrix* sp.; average position is x = 0.2941±0.0632, y = 0.4821±0.2900. *Lineus alborostratus*; average position is x = 0.2729±0.0435, y = 0.7299±0.0588. *Lineus virids*; average position is x = 0.1800±0.0533, y = 0.7734±0.1243. *Zygeupolia rubens*; average position is x = 0.1902±0.0597, y = 0.9176±0.0701. *Emplectonema gracile*, average position is x = 0.2288±0.0430, y = 0.7031±0.1273. *Nectonemertes* cf. *mirabilis*; average position is x = 0.1433±0.0432, y = 0.7752±0.1114. *Paranemertes* cf. *peregrina*; average position is x = 0.2270±0.0695, y = 0.8814±0.0814.

To investigate whether these biased codon choices are restricted in highly biased protein-coding genes, the association between purines (A and G) and pyrimidines (C and T) in four-codon amino acid families was analyzed by Parity Rule 2 (PR2) bias plot [32] (Figure 3). G and T were more frequent than C and A in all comparisons except in two *Cephalothrix* species (Figure 3). Differences between C and G and between A and T contents were observed commonly in most protein-coding genes.

The wobble nucleotides of tRNA anticodons in nemertean mitochondrial genomes show a strong bias towards G and U, which is congruent with the mutation bias of the coding strand.

In comparing tRNA anticodon and synonymous codon families (Table 3), except for stop codons, we found seven of the NNY codon family (where N represents any of the four nucleotides and Y either C or U) are “non-optimal codon–anticodon usage” [37]. Each with one synonymous codon, whose corresponding tRNA is not encoded in the mtDNA, used strongly preferred. The optimal codons ended with U, such as UGU for Cys. The RSCU value of UGU was higher than for UGC, yet the wobble position of the tRNA-Cys anticodon was G. Also, “combined codon–anticodon usage” is found in the NNN codon family; two synonymous codons are used substantially more preferentially and corresponding tRNA is both encoded and not encoded in the mtDNA. Both of the two codon RSCUs were distinctly higher than the other two codons in the four-fold synonymous codon families. For example, the RSCU values of ACA and ACU, which encode Thr, were the highest, but the wobble position of tRNA- Thr anticodon was only U. Thus, only ACA could completely pair with the anticodon.

Codon usage also can be influenced by translational selection. Because tRNAs are involved in protein translation, there is a high correlation between preferred codons and the abundance of corresponding tRNAs in many bacteria [1], [38]. Only one tRNA gene translates a synonymous codon family in animal mitochondrial genomes. Nevertheless, it is still possible for translational selection to occur between synonymous codons translated by a single tRNA, if one codon interacts more effectively than another with the anticodon of tRNA [33]. The codon-anticodon adaptation hypothesis described for vertebrate mitogenomes by Xia [36] predicts that the anticodon should match the most abundant codon. Our analysis of the RSCU and tRNAs encoded by the mtDNA shows that most NNY and NNN family codons could not pair completely with the tRNA anticodons in nemertean mitochondrial genomes. Thus, our findings from nemerteans do not support the selection hypothesis of anticodon adaptation, which also was refuted by studies on bivalves, arthropods and fungi [39], [40], [41].

### Context-dependent mutation in nemertean mitogenomes

To investigate the complexity of mutational dynamics in nemertean mitogenomes, data of the nine FFD families were analyzed. The independence of nucleotides at the second and third codon positions was analyzed from three datasets (Table 4): Leu/Pro/Arg; Val/Ala/Gly; and (Leu+Val)/(Ser1+Pro+Thr+Ala)/(Arg+Gly+Ser2). Each of these three datasets forms a 3×4 contingency table, and was tested by a χ^2^ test for independence between nucleotide frequencies at the third codon position (codon usages) and second codon position (U, C or G) (Table 4).

**Table 4:** Independence test (data shown as *P*-values of *x* ^2^ tests) between the second and third codon positions.

Amino acids	Bases of codon positions		*Cephalothrix hongkongiensis*	*Cephalothrix* sp.	*Lineus alborostratus*	*Lineus viridis*	*Zygeupolia rubens*	*Emplectonema gracile*	*Nectonemertes* cf. *mirabilis*	*Paranemertes* cf. *peregrina*
	1st	2nd, 3rd								
L/P/R	C	UN/CN/GN	<0.0001	0.0031	0.0008	0.0004	0.0007	0.0072	0.0039	<0.0001
V/A/G	G	UN/CN/GN	<0.0001	<0.0001	<0.0001	<0.0001	<0.0001	<0.0001	<0.0001	<0.0001
LV/S1PTA/RGS2	S/N/S	UN/CN/GN	<0.0001	<0.0001	<0.0001	<0.0001	<0.0001	<0.0001	<0.0001	<0.0001

Evidence of context-dependent mutation has previously been reported from both animal and plant mitochondrial genomes [33], [34]. The relative frequencies of the four codons in each FFD family should be similar, if the frequency of the nucleotide at the third codon positions is not influenced by other sites. This expectation is not borne out in our results (Table 4). Based on three different datasets and the *x* ^2^ tests with 6 degrees of freedom, the calculated *P*-values were extremely significant in all cases, suggesting that the base at the second codon position affected the synonymous codon choices.

**Figure 4:**
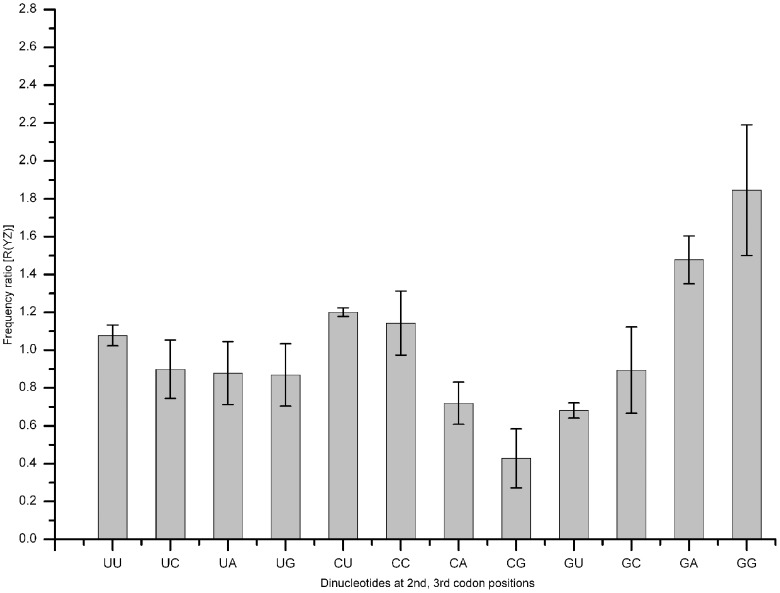
Frequency ratios [R(YZ)] of 12 dinucleotides at second and third codon positions. Values are averaged from eight nemertean species.

Among all of the nine FFD families, the nucleotide at the second codon position (Y in a given codon XYZ) could be U, C, or G, and the nucleotide at the third codon position (Z in a given codon XYZ) could be any of the four regular bases. Context-dependent mutational processes create correlations between neighboring bases. These can be measured by dinucleotide frequency ratio *R*(YZ) defined as *f*_YZ_/(*f*_Y_ *f*_Z_), as in a previous study [33]. For a total of 12 possible dinucleotides, *R*(YZ)s were calculated using all three datasets for each nemertean mitochondrial genome (Figure 4). The calculated frequency ratios did not deviate much from 1 for most dinucleotides, but there are some exceptions. When the nucleotide is G at the second codon position, the nucleotide at the third codon position is more likely to be G, with mean *R*(GG) in eight nemertean mitochondrial genomes equal to 1.84±0.34, which is a statistically significant deviation from the expected ratio 1. When the nucleotide is C at the second codon position, the nucleotide G is less likely to be at the third codon position, with mean *R*(CG) equal to 0.43±0.16, reflecting a prejudice against NCG codons.

Jia and Higgs [33] had previously reported *R*(YZ) ratios based on mammal mitochondrial data. The nemertean mitochondria showed similar ratios for the dinucleotides. The largest ratio is R(GG) (nemertean to mammal: 1.84±0.34 *vs* 1.65, whereas the smallest is R(CG) (0.43±0.16 *vs* 0.55) in all cases. These results suggest that there are context-dependent mutational effects influencing dinucleotide positions in a similar way in both nemerteans and mammals.

Relative dinucleotide frequencies in vertebrate mitochondrial genomes are also reported by [42]. They consider all pairs, irrespective of reading frame or whether the sequence is coding or noncoding. They also observe GG is most overrepresented and CG is most underrepresented. One process known as ‘CpG effect’ showed that CG dinucleotide is a mutational hotspot in mammalian genomes [5], which may explain a low frequency ratio of R(CG) in animal mitogenomes.

## Conclusion

This study conclusively demonstrates that the genome-wide codon usage bias in nemertean mitochondrial genomes is mainly set by mutational force, though the dynamics may be complex. Codon usage of the nemertean mitochondrial genomes appears to be not necessarily constrained by tRNA anticodons, suggesting that codon usage and anticodons evolve independently. Therefore, the mutation together with selection dynamics may play an important role in shaping the pattern of codon usages in nemertean mitochondrial genomes. Different genomes have their own characteristic patterns of synonymous codon usage [43]. The previous studies on genomes of *Drosophila* [44] and nematodes [45], [46] demonstrated that codon usage similarity usually persists over the breadth of a genus but then rapidly diminishes within each clade of higher taxonomic rank. However, Ingvarsson [47] showed that five species within the genus *Populus* with close phylogenetic relationship have significantly different synonymous codon bias. Therefore, it may be difficult to draw conclusions about assumed codon usage patterns to unsampled species beyond the genus level. Additionally, different life history characteristics can potentially influence effective population size which will affect patterns of codon usage (e.g., [48]). This might be the same phenomenon in nemerteans, for instance, palaeonemertans and hoplonemerteans have direct development, and heteronemerteans have indirect development with larval stages. Nevertheless, basic knowledge of codon usage patterns of nemertean mitochondrial genomes and the factors regulating the codon usage are presented in the study, more comprehensive analysis is necessary for revealing the deeper characteristic of codon usage, when more nemertean mitochondrial genomes are available.
